# Genetic analysis of salinity tolerance in wheat (*Triticum aestivum* L.)

**DOI:** 10.1371/journal.pone.0265520

**Published:** 2022-03-17

**Authors:** Saeed Omrani, Ahmad Arzani, Mohsen Esmaeilzadeh Moghaddam, Mehrdad Mahlooji

**Affiliations:** 1 Department of Agronomy and Plant Breeding, College of Agriculture, Isfahan University of Technology, Isfahan, Iran; 2 Seed and Plant Improvement Institute (SPII), Agricultural Research, Education and Extension Organization (AREEO), Karaj, Iran; 3 Isfahan Agricultural and Natural Resources Research and Education Center (AREEO), Isfahan, Iran; Institute of Genetics and Developmental Biology Chinese Academy of Sciences, CHINA

## Abstract

Understanding the genetics of salt tolerance is of utmost need to combat the rising prevalence of soil salinity through employing tolerant cultivars. The current study was carried out to investigate the quantitative genetic basis of agronomical and physiological-related traits of salinity-stressed plants using seven generations (parental cultivars, F_1_, F_2_, F_3_, BC_1_, and BC_2_) of wheat grown in the field under normal and saline conditions. The combined analysis of variance showed highly significant effects of salinity and genotypes (generations) on all the traits. The scaling tests did not support the three-parameter model (additive-dominance model); hence, the six-parameter model was used to assess the genetic effects governing the traits in this study. The epistatic gene effects were crucial, as were additive and dominance gene effects for plant height, K/Na, and yield in salinity stress conditions. The highest heritability was observed for total chlorophyll, carotenoid, SPAD chlorophyll, and K/Na ratio in saline conditions. The additive genetic variance was more important than the dominance variance for grain weight, K, K/Na in salinity conditions. The findings of the current study may have important implications in the quantitative genetics of salinity tolerance and the development of cultivars tolerant to salinity in wheat.

## Introduction

Common wheat (*Triticum aestivum* L.) is a crucial staple food with global production of over 700 million tones, contributes significantly to the diet of the world population by supplying 20% of protein and daily calories to 4.5 billion people worldwide [1]. Saline soils are one of the abiotic factors that have adversely affected plant production, especially in the arid and semi-arid regions of the world [2]. Iran is a region where the far-most western side of the Fertile Crescent, as the center of origin of wheat (*Triticum* spp.) [1], is one of the countries with an estimated 18 to 27 million hectares of saline lands has been affected by salinity to various degrees.

The adverse effects of salinity on plant growth and development can be implemented in two phases according to the classical view [2, 3]. In the first phase, osmotic stress happens immediately after exposure to salinity stress and inhibits plant growth. At the second phase, ionic stress (toxicity) happens when plant is exposed to salt for several days or weeks depending on the severity of salinity stress and when toxic ions (e.g., Na^+^ and Cl^−^) accumulate to high concentrations beyond the plant-specific thresholds in the leaves. Plants that tolerate osmotic stress can be maintained their growth rate in the initial stage of salinity exposure [4]. Therefore, in addition to osmoregulation/osmotic adjustment, enhancing osmotic stress tolerance may take place by two opposing strategies. The selection of plants with low leaf area represents the first approach to improve stomata performance. Conversely, selecting of plants with more leaf area and capacity to intercept light is the second strategy to improve the required energy for water uptake by the roots and water flow through the plant [3].

Salinity tolerance is a complex polygenic trait highly influenced by environmental factors and genetic-environment interaction, but we do not yet fully understand the genetic architecture [2]. Generation mean analysis is a reliable biometrical genetic approach in dissecting gene effects in a quantitative trait by having an additional benefit of subtracting the variances of digenic genetic interactions (additive × additive [i], additive × dominance [j], and dominance × dominance [l]) [5]. It is not only critical to exploit natural variations in wheat for saline adaptation but also crucial to understand in terms of their genetic mode of action and implementation in a breeding program.

Although there are some studies on the inheritance of salinity tolerance in crop plants, most of them were done with young plants cultivated in pots under greenhouse conditions (see [6]). Koch et al. [7] investigated the inheritance of yield in salinity tolerance in ryegrass using a diallel design with six parental clones. They found relatively high narrow-sense heritability estimates for salinity tolerance. In durum wheat, Shamaya et al. [8] used F_2_ populations of durum wheat to assess the broad-sense heritability of leaf Na content and the K/Na ratio of hydroponically-grown plants exposed to 100 mM NaCl at the seedling stage under greenhouse conditions. They reported a high broad-sense heritability for shoot Na^+^ concentration. In another study by Munns and coworkers [9] a moderate to high heritability was seen for this trait in two F_2_s of durum wheat. Notably, the results of a pot experiment in common wheat suggested the importance of epistatic effects in governing salinity tolerance [10]. Salinity tolerant plant maintains a low concentration of Na in the cytosols of root and shoot cells through Na exclusion, extrusion or compartmentation under saline conditions [11]. In addition, maintenance of a high concentration of K in the cytosols help plant to minimize salinity damage under salinity stress. Therefore, the K/Na ratio in the root and shoot is an important variable when studying salinity tolerance in many plant species, including wheat [12] and barley [13, 14].

Despite the large body of data on the genetic studies carried out in non-saline field conditions [15–18], little has been published on the inheritance of salinity tolerance in wheat plants grown under field conditions. The current study aimed to investigate the genetic basis of salinity tolerance in wheat using the agronomical and physiological performances of seven generations (parental cultivars, F_1_, F_2_, F_3_, BC_1_, and BC_2_) grown in normal and saline field conditions.

## Materials and methods

### Plant materials

A salt tolerant (‘Barat’) and a salt-sensitive (‘Nogal’) wheat cultivar were crossed to produce the following filial and backcross progenies: F_1_, F_2_, F_3_, BC_1_, and BC_2_, at Research Farm of Isfahan Agricultural and Natural Resources Research and Education Center. ‘Barat’ cultivar has a pedigree of SLVS*2/PASTOR, was selected from 24th SAWSN of International Maize and Wheat Improvement Center (CIMMYT), and released in the warm and dry areas of South Iran [19]. This cultivar is tolerant to salinity stress (unpublished data) [19]. In contrast, ‘Nogal’ appears to be a salinity-sensitive cultivar [20] and is a widely-grown modern wheat cultivar in Europe [21]. This cultivar is also sensitive to water deficit conditions explaining that not selected for tolerance to stress conditions [22].

### Field experimental and salinity stress conditions

Two parental cultivars and their five derived populations (seven generations) were planted in normal and salt-stress experiments using a randomized complete block design with three replicates. Each replicate or block consisted of 194 rows including two rows for parents (P_1_ and P_2_) and F_1_, four rows for F_2_, BC_1_, BC_2_, and one row for each of the 176 F_3_ families. The rows were 1.5 m long and 30 cm apart with 5 cm between plants (30 plants/row). The seeds of the parents and progenies were handplanted in the rows within a replication. The plants in the normal experiment were irrigated with freshwater (electrical conductivity (EC_w_ = 0.8 dS m^−1^), while in the salinity-stress experiment were irrigated with brackish groundwater with the EC of 13 dS m^−1^ (mainly of NaCl type salt). The saline irrigation was applied eight times, starting from the four-leaf stage (Zadoks growth stage 14) to ripening stage. No precipitation was recorded during the salinity treatment period. Soil samples were taken from the 0 to 30 cm depth to determine the EC of saturated soil–paste extracts (ECe) at the beginning and just after harvest. The ECe was determined following the Rhoades [23] method using an EC meter with a saturation extract of 1:5 soil to water ratio. Deionized water was used for this measurement. Three soil samples from each experimental plot (generation within a replicate) were used. An average EC_e_ within the root zone (30 cm) for the normal and saline field conditions was determined as 1.8 and 9.6 dS m^−1^, respectively. Based on soil sample analysis, 30 kg P ha^−1^ and 120 kg N ha^−1^ fertilizers were applied before sowing. Nitrogen fertilizer was splitted into three parts and applied at sowing, tillering, and anthesis stages.

### Agronomical traits

The following variables were measured: number of days to heading date (DH), number of days to pollination date (DP), number of days to maturity date (DM), plant height (PH), peduncle length (PL), single grain weight (GW), number of spikes per plant (NS), number of grains per spike (GN), grain yield per plant (yield). To measure the agronomical traits, samples were obtained from 15 plants of each P_1_, P_2_, and F_1_, 30 plants of each BC, 50 plants of F_2_, and 10 plants from each F_3_ family per replicate (see S1 Table).

#### Physiological traits

To measure the physiological traits, leaf samples were obtained from 10 plants of each P_1_, P_2_, and F_1_, 20 plants of each BC and F_2_, and five plants from each F_3_ families per replicate (see S2 Table).

#### Na and K concentration

Leaf samples (100 mg) were incinerated at 550°C for 4 h. Inorganic ions were then extracted using 10 mL HCl (2 N), and the volume of each sample was standardized to 100 ml. The sodium and potassium contents of the solutions were determined by flame photometry (Jenway PFP7, UK). A standard curve was used to determine the Na and K concentrations [12]. The K/Na ratio was then calculated.

#### Relative water content (RWC)

A sample of fresh leaves was weighed (FW) immediately after cutting into pieces, placed in 20 mL of distilled water, and left in the dark for 24 h at room temperature. Then the weight of turgid leaves was used as turgid weight (TW). The oven-dried (70°C for 48 h) samples were weighted to determine the dry weights (DW). The total RWC was then determined using the below formula [14]:

%RWC=[(FW‐DW)/(TW‐DW)]×100

#### Leaf chlorophyll and carotenoid concentrations

Chlorophyll a, Chlorophyll b and total carotenoid concentrations were measured using the spectrophotometer method (Hitachi F-2500 fluorescence spectrophotometer) with reading absorbance at 646, 663, and 470 nm, respectively. To determine photosynthetic pigments, 0.1 g fresh leaf tissue sampled at early heading stage (Zadoks growth stage 58) were homogenized and extracted in 15 mL acetone 80% using a centrifuge at 5000 rpm for 15 min. Chlorophylls (a and b) and carotenoids (Car) concentrations (mg/g FW) were calculated using below equations [24]:

$$Chlorophyll\;a=[(13.36\times A_{663})‐(5.19\times A_{646})\times 8.1]/FW$$

$$Chlorophyll\;b=[(27.43\times A_{646})‐(8.12\times A_{663})\times 8.1]/FW$$

$$Carotenoids=[(4.785\times A_{470})+(3.657\times A_{663})‐(12.76\times A_{646})\times 8.1]/FW$$

Total chloroyll (Tchl) was calculated as the sum of chlorophyll a and b. Only Tchl and Car were subjected to genetic analysis.

#### Flag leaf SPAD value and area

Flag leaf blade chlorophyll (i.e., SPAD reading) was estimated using three randomly selected flag leaves from each plot by a SPAD chlorophyll meter (SPAD-502, Konica Minolta, Japan). The measurements were conducted at the early heading stage (Zadoks growth stage 58). Flag leaf area (FLA) was measured by a leaf area meter. The measurements were carried out at the full heading stage (Zadoks growth stage 60).

### Statistical analysis

The data were initially checked for normality of distribution and homogeneity of variance using Kolmogorov–Smirnov and Bartlett’s tests, respectively. A combined analysis of variance (ANOVA) was used to test the effects of the environment (normal and salinity), generation, and generation × environment interaction by mixed-model ANOVA using the Mixed Procedure of SAS. Replicate and environment were considered fixed factors, and generation was random. The statistical analyses were carried out using the SAS software package (SAS, Institute, Cary, NC, USA) and SPSS (version 26). Tukey’s Honest Significant Difference (HSD) test was used to compare trait means obtained from each set of the seven generation groups (two parents and five progenies).

### Genetic analysis

Generation mean analysis was conducted following Mather and Jinks [5] model as below:

$$Y=m+a[d]+\beta [h]+a^{2}[i]+2a\beta [j]+\beta^{2}[l]$$

Where Y is the mean of phenotypic value of plant individuals in each generation; [d], [h], [i], [j] and [l] are the effects of genetic parameters (additive, dominance, additive × additive epistasis, additive × dominance epistasis and dominance × dominance epistasis, respectively); and α, β, α^2^, 2αβ and β^2^ are the coefficients of these genetic parameters, respectively. A weighted least-squares analysis was used following the model originally developed by Cavalli [25] and extended by Hayman [26] to obtain the genetic effects for each trait. The analysis was conducted using the means of seven generations, weighted by the reciprocal of the variance of each generation mean (Qi/Vi), where Qj = the mean of the i^th^ generation and Vi = the variance of the i^th^ generation mean.

Mather’s scaling test A, B, C and D was initially conducted and then followed by the Joint scaling test of Cavalli [25] to examine the goodness-of-fit of the additive-dominance model. The standard error of the calculated scales (A, B, C and D) was t-tested for significance. On the other hand, the absence of epistasis can be confirmed by chi-square (χ^2^). The best fit model is the one with a non-significant chi-square and significant estimates of the scales. If the results point out the role of epistasis, then a six-parameter genetic model (m, d, h, i, j, and l) should be fitted to the generation means [27]. Broad-sense and narrow-sense heritability were estimated following the procedures outlined by Warner [28]:

$$h^{2}{}_{b}=100\times [V_{F2}–(V_{P1}+V_{P2}+2V_{F1})/4]/V_{F2}$$

$$h^{2}{}_{n}=100\times [2–VF2–(V_{BC1}+V_{BC2})]/V_{F2}$$

Components of genetic variance were calculated based on the data of six generations using the relationships suggested by Mather and Jinks [5] as follows:

$$V_{E}{}_{}=(V_{P1}+V_{P2}+V_{F1})/3$$

$$F=V_{BC1}-V_{BC2}$$

$$D=4V_{F2}-2(V_{BC1}+V_{BC2})$$

$$H=4\;(V_{BC1}+V_{BC2}-V_{F2}-V_{E}),$$

Where, V_E_ = environmental variance; D = additive variance; H = dominance variance; V_F2_, V_F1_, V_BC1_, V_BC2_, V_P1_ and V_P2_ are variance of F_2_, F_1_, BC_1_, BC_2_, P_1_ and P_2_, respectively; and F = joint contribution on all loci.

## Results

### Agronomical traits

The results of combined-ANOVA for the traits studied in both normal and salinity stress conditions are presented in Table 1. In this study the numbers of plants per replication were almost identical and were consistent for both experiments. The separate analysis of variance for the generations in each environment revealed that the replication and interaction between generation × replication did not approach significance. Therefore, generation means analysis was conducted without adjusting the data for replication. There were significant effects of both salinity and genotypes (generations) on all the traits. The genotype × environment interaction for most of the traits except for PH, PL, FLA, DH, DP, DM was significant. Given the existence of genetic variation in the tolerance to salinity, genetic analysis can be undertaken to estimate different genetic parameters such as components of genetic variance and the heritability of salinity tolerance.

**Table 1:** Results of combined analysis of variance of agronomical and physiological traits studied in seven generations under normal and salinity-stress field conditions.

Source of variation	Df	Mean square								
		PH	PL	FLA	GW	DH	DP	DM	NS	GN
Salinity (S)	1	244.44**	5.08**	1665.51**	0.74**	39.29**	112.23**	132.46**	50.20**	509.50**
Rep (Salinity)	4	15.32	0.04	0.21	0.01	16.07	13.16	10.39	0.01	0.62
Genotype (G)	6	42.63**	7.27**	998.34**	0.10**	30.23**	26.75**	21.58**	1.95**	72.40**
S × G	6	0.10^ns^	0.06^ns^	120.33^ns^	0.06**	0.90^ns^	1.12^ns^	1.29^ns^	0.16*	13.67**
Residual	24	3.23	0.22	73.42	0.01	2.44	1.26	1.83	0.06	2.06
CV (%)		8.14	10.06	15.23	12.17	4.18	2.33	3.15	8.92	9.04
Source of Variation	df	Yield	RWC	Na	K	K/Na ratio	SPAD	Tchl	Car	
Salinity (S)	1	105.61**	234.09**	0.01**	1.07**	730.53**	1.97**	0.78**	0.03**	
Rep (Salinity)	4	8.83	5.87	0.07	0.74	18.46	0.07	0.32	0.12	
Genotype (G)	6	99.44**	18.82**	0.01**	0.03**	355.27**	19.53**	0.01**	0.01**	
S × G	6	11.48**	8.58**	0.01**	0.02**	278.16**	2.32**	0.01**	0.01**	
Residual	24	1.23	0.03	0.01	0.02	0.01	0.14	0.01	0.04	
CV (%)		17.82	6.77	21.17	18.74	15.43	4.42	15.71	9.46	

* and **: statistically significant at *p* < 0.05 and < 0.01, respectively.

PH, PL, FLA, GW, DH, DP, DM, NS, GN, Yield, RWC, Na, K, K/Na, SPAD, Tchl, and Car denote; plant height, peduncle length, flag leaf area, grain weight, number of days to heading, number of days to pollination, number of days to maturity, number of spikes per plant, number of grains per spike, grain yield per plant, relative water content, SPAD chlorophyll, total chlorophyll, and carotenoid concentration, respectively.

‘Barat’ exhibited higher PL and FLA than ‘Nogal’ but had lower yield and RWC in both environmental conditions (Tables 2 and 3). In addition, ‘Barat’ performed better than another parent in terms of GW and GN in salinity stress conditions (Table 3). At the same time, the highest mean values of the traits mentioned above were obtained by ‘Nogal’ in normal conditions (Tables 2). ‘Barat’ had a lower rate of Na accumulation and a higher K/Na discrimination compared with ‘Nogal’ in both treatments. The GW, NS, and yield in F_1_ were higher than those of the two parents in normal and salinity stress conditions. The F_1_ values for PL, DH, DM, SPAD were almost intermediate between those of the parents in both treatments. The mean of trait values in the F_2_ generation was lower than the corresponding values for the F_1_ generation (Tables 2 and 3). Furthermore, generations differed in GW, GN, and Car within each environment and did respond differently to the two environmental conditions (Tables 2 and 3).

**Table 2:** Mean comparisons of the traits studied in seven generations of wheat under normal conditions.

Generation	PH	PL	FLA	GW	DH	DP	DM	NS	GN
	(cm) (cm)		(cm^2^)	(g)					
P_1_	76.86 ^a^*	10.53 ^a^	151.20 ^a^	0.036 ^c^	173.19 ^c^	175.33 ^d^	217.51 ^bc^	9.02 ^b^	40.61 ^c^
P_2_	67.59 ^c^	6.44 ^d^	103.61 ^cd^	0.039 ^ab^	180.40 ^a^	189.67 ^a^	223.63 ^a^	8.47 ^cd^	48.70 ^a^
F_1_	73.41 ^b^	7.94 ^c^	118.34 ^c^	0.040 ^a^	176.13 ^bc^	186.64 ^b^	219.58 ^b^	9.69 ^a^	45.19 ^ab^
F_2_	70.25 ^bc^	7.37 ^cd^	104.39 ^cd^	0.039 ^ab^	175.06 ^bc^	182.34 ^bc^	217.65 ^bc^	8.24 ^cd^	37.61 ^d^
F_3_	67.79 ^c^	7.11 ^cd^	95.74 ^d^	0.035 ^d^	172.88 ^c^	181.22 ^bc^	216.14 ^c^	7.77 ^d^	35.77 ^e^
BC_1_	71.18 ^bc^	8.76 ^b^	131.63 ^b^	0.036 ^cd^	175.08 ^bc^	177.01 ^c^	218.70 ^bc^	8.60 ^c^	42.16 ^bc^
BC_2_	67.95 ^c^	6.82 ^cd^	100.21 ^cd^	0.038 ^b^	177.93 ^b^	181.11 ^bc^	220.28 ^b^	8.49 ^cd^	44.41 ^b^
Generation	Yield	RWC	Na	K	K/Na Ratio	SPAD	Tchl	Car	
	(g m^-2^)	(%)	(mg g^-1^ DW)				(mg g^-1^ FW)	
P_1_	24.41 ^c^	83.65 ^f^	0.058 ^d^	1.87 ^a^	38.40 ^c^	48.33 ^d^	0.64 ^f^	0.15 ^c^	
P_2_	38.07 ^a^	86.82 ^a^	0.129 ^a^	1.79 ^e^	14.89 ^f^	53.29 ^a^	0.66 ^d^	0.14 ^e^	
F_1_	38.50 ^a^	84.81 ^b^	0.108 ^b^	1.84 ^b^	17.37 ^e^	49.06 ^cd^	0.60 ^g^	0.14 ^f^	
F_2_	24.58 ^c^	84.25 ^c^	0.058 ^e^	1.81 ^c^	5.34 ^g^	47.41 ^e^	0.69 ^a^	0.17 ^a^	
F_3_	24.03 ^c^	83.41 ^g^	0.060 ^c^	1.60 ^g^	40.86 ^a^	47.36 ^e^	0.67 ^c^	0.16 ^b^	
BC_1_	26.85 ^c^	83.91 ^e^	0.051 ^g^	1.81 ^d^	38.66 ^b^	49.67 ^c^	0.65 ^e^	0.14 ^g^	
BC_2_	31.44 ^b^	84.04 ^d^	0.056 ^f^	1.66 ^f^	34.34 ^d^	51.50 ^b^	0.69 ^b^	0.14 ^d^	

PH, PL, FLA, GW, DH, DP, DM, NS, GN, Yield, RWC, Na, K, K/Na, SPAD, Tchl, and Car denote; plant height, peduncle length, flag leaf area, grain weight, number of days to heading, number of days to pollination, number of days to maturity, number of spikes per plant, number of grains per spike, grain yield per plant, relative water content, SPAD chlorophyll, total chlorophyll, and carotenoid concentration, respectively.

* Values followed by different letters in each column are significantly different (*p* < 0.05) using Tukey’s HSD.

**Table 3:** Mean comparisons of the traits studied in seven generations of wheat under salinity-stress field conditions.

Generation	PH	PL	FLA	GW	DH	DP	DM	NS	GN
	(cm)	(cm)	(cm^2^)	(g)					
P_1_	69.35 ^a^*	9.45 ^a^	116.04 ^a^	0.037 ^a^	170.83 ^cd^	172.22 ^d^	213.28 ^b^	12.41 ^a^	37.81 ^a^
P_2_	61.74 ^c^	5.90 ^d^	92.37 ^bc^	0.031 ^c^	178.64 ^a^	183.71 ^a^	217.33 ^a^	10.88 ^c^	34.14 ^b^
F_1_	68.88 ^ab^	7.13 ^bc^	112.03 ^ab^	0.037 ^a^	174.36 ^bc^	176.36 ^ab^	216.16 ^ab^	12.51 ^a^	36.53 ^ab^
F_2_	63.62 ^c^	6.38 ^c^	95.08 ^b^	0.035 ^ab^	172. 92 ^c^	175.28 ^b^	212.49 ^c^	10.66 ^cd^	28.16 ^c^
F_3_	62.86 ^c^	5.95 ^d^	80.68 ^c^	0.032 ^c^	171.25 ^cd^	174.45 ^bc^	211.59 ^c^	10.55 ^d^	25.48 ^c^
BC_1_	65.14 ^b^	7.92 ^b^	106.33 ^ab^	0.035 ^ab^	170.69 ^d^	172.36 ^d^	214.79 ^ab^	10.76 ^cd^	36.60 ^ab^
BC_2_	62.06 ^c^	6.28 ^cd^	94.63 ^bc^	0.034 ^b^	175.41 ^b^	173.16 ^c^	217.40 ^a^	11.26 ^b^	36.03 ^ab^
Generation	Yield		RWC	Na	K	K/Na Ratio	SPAD	Tchl	Car
	(g m^-2^)		(%)	(mg g^-1^ DW)				(mg g^-1^ FW)	
P_1_	21.02 ^c^		73.47 ^g^	0.09 ^c^	1.42 ^b^	16.52 ^d^	47.61 ^c^	0.98 ^d^	0.20 ^g^
P_2_	29.11 ^a^		80.59 ^a^	0.18 ^a^	1.36 ^e^	7.84 ^f^	53.61 ^a^	1.10 ^a^	0.23 ^c^
F_1_	29.98 ^a^		80.02 ^b^	0.15 ^b^	1.42 ^d^	10.01 ^e^	50.05 ^b^	1.03 ^c^	0.20 ^f^
F_2_	23.25 ^b^		79.34 ^c^	0.08 ^f^	1.40 ^c^	20.97 ^b^	47.54 ^c^	0.97 ^e^	0.22 ^b^
F_3_	22.24 ^c^		77.03 ^e^	0.08 ^e^	1.28 ^g^	6.16 ^g^	47.84 ^c^	0.97 ^f^	0.22 ^d^
BC_1_	25.17 ^b^		75.66 ^f^	0.08 ^d^	1.43 ^a^	19.67 ^c^	46.22 ^d^	0.97 ^g^	0.22 ^e^
BC_2_	29.91 ^a^		79.79 ^d^	0.07 ^g^	1.31 ^f^	37.17 ^a^	50.04 ^b^	1.06 ^b^	0.25 ^a^

PH, PL, FLA, GW, DH, DP, DM, NS, GN, Yield, RWC, Na, K, K/Na, SPAD, Tchl, and Car denote; plant height, peduncle length, flag leaf area, grain weight, number of days to heading, number of days to pollination, number of days to maturity, number of spikes per plant, number of grains per spike, grain yield per plant, relative water content, SPAD chlorophyll, total chlorophyll, and carotenoid concentration, respectively.

* Values followed by different letters in each column are significantly different (*p* < 0.05) using Tukey’s HSD.

### Goodness-of-fit of the genetic model

The results of scaling tests can be used to test the hypothesis of the goodness of fit of the three-parameter model (additive-dominance model) or non-significant effect of epistasis (non-allelic interaction). The results of both individual and joint scaling tests summarized in Table 4 did not support the hypothesis of the additive-dominance model for all the traits in both environmental conditions. In addition, the majority of the traits showed more than one significant scale, indicating the combined effects of epistasis. The six-parameter model was subsequently utilized to assess the genetic effects governing the traits in this study.

**Table 4:** Results of individual and joint scaling tests for the traits studied in seven generations of wheat under normal and salinity-stress field conditions.

Trait	Normal					Salinity stress				
	A	B	C	D	χ^2^	A	B	C	D	χ^2^
PH	-4.55** ± 0.38	1.35^ns^ ± 0.24	-14.89** ± 1.33	-11.07** ± 0.47	130.4**	-5.73** ± 0.34	-2.2^ns^ ± 0.08	-8.24** ± 0.19	-7.94** ± 0.24	81.4**
DH	-0.97** ± 0.21	1.95**^`^± 0.39	-9.89** ± 0.73	-19.31** ± 1.88	93.3**	-4.55** ± 0.16	1.38* ± 0.03	-13.57** ± 0.42	-9.05* ± 0.15	119.9**
DP	4.14** ± 0.48	0.21^ns^± 0.04	-7.9*± 0.45	-3.84* ± 0.37	33.7**	-5.86** ± 0.03	-15.67** ± 0.53	-8.53** ± 0.17	-13.73** ± 0.42	56.7**
DM	-0.25^ns^ ± 0.04	-2.72* ± 0.43	-12.12** ± 1.06	-8.54** ± 0.63	25.7**	0.93*± 0.05	4.01* ± 0.13	-9.91** ± 0.48	-6.61** ± 0.19	44.8**
PL	-0.85^ns^ ± 0.12	-0.77* ± 0.21	-2.22** ± 0.09	-2.1** ± 0.37	22.6**	-1.58** ± 0.09	1.01* ± 0.11	-6.07* ± 0.31	-5.19** ± 0.52	33.4**
FLA	0.04^ns^ ± 0.01	3.09** ± 0.41	-0. 04^ns^ ± 0.01	0. 03^ns^ ± 0.01	27.3**	-2^ns^ ± 0.12	9.67* ± 0.23	-0.03^ns^ ± 0.01	0.04^ns^ ± 0.01	31.7*
GW	-0.44** ± 0.05	-0.07^ns^ ± 0.01	-0.63* ± 0.02	-0.87** ± 0.03	14.2**	-0.26^ns^ ± 0.02	0.06^ns^ ± 0.01	-0.07^ns^ ± 0.01	-0.89* ± 0.11	38.48*
NS	-2.95^ns^ ± 0.39	0.43^ns^ ± 0.11	-5.84* ± 0.28	-3.2^ns^ ± 0.16	31.7*	-3.37** ± 0.12	1.02* ± 0.04	-6.05** ± 0.44	-2.09* ± 0.18	92.3**
GN	-5.54** ± 0.14	4.1* ± 0.33	-23.96** ± 1.73	-18.88** ± 0.88	31. 2**	0.11^ns^ ± 0.01	6.5** ± 0.28	-31.99** ± 1.13	-24.43** ± 1.48	54.8**
Yield	-10.12** ± 0.58	0.82* ± 0.07	-37.82** ± 1.48	-15.5** ± 0.78	62.1**	-3.16** ± 0.33	5.17* ± 0.14	-16.11** ± 1.03	-7.11** ± 0.24	92.6**
RWC	-1.44* ± 0.17	-4.72** ± 0.23	-2.76*± 0.02	-6.2** ± 0.37	29.3**	-1.21^ns^ ± 0.08	-7.74^ns^ ± 0.31	17.1* ± 0.46	-16.04^ns^ ± 0.55	32.3*
SPAD	-6.09** ± 0.14	-0.44^ns^ ± 0.03	-10.59** ± 0.72	-5.39** ± 0.21	18.4**	-0.05*± 0.01	1.41*± 0.06	-9.65** ± 0.17	-7.75** ± 0.33	71.7**
Na	-0.05* ± 0.01	0.08*± 0.01	-0.14^ns^ ± 0.06	-0.07** ± 0.01	22.6**	-0.07^ns^ ± 0.01	-0.11* ± 0.02	-0.18* ± 0.08	-0.10** ± 0.01	64.2**
K	-0.13^ns^ ± 0.08	-0.14* ± 0.02	-0.39** ± 0.06	-0.71** ± 0.02	37.1**	-0.22** ± 0.02	-0.28^ns^ ± 0.01	0.03*± 0.01	-0.69** ± 0.08	36.9**
K/Na	-2** ± 0.11	-8.56** ± 0.13	6.28** ± 0.41	-17.12** ± 0.31	84.7**	-0.2* ± 0.02	-1.01** ± 0.11	0.05^ns^ ± 0.01	0.04* ± 0.01	22.2**
Tchl	-0.04^ns^ ± 0.01	-0.03* ± 0.01	-0.03^ns^ ± 0.01	0.04^ns^ ± 0.01	19.2**	0.02* ± 0.01	-0.03* ± 0.01	-0.04* ± 0.01	-0.05^ns^ ± 0.01	24.9**
Car	-0.05^ns^ ± 0.01	-0.04^ns^ ± 0.01	0.22** ± 0.02	0.04^ns^ ± 0.01	21.9**	0.02** ± 0.01	0.03^ns^ ± 0.01	0.06^ns^ ± 0.01	-0.05* ± 0.01	47.7**

PH, PL, FLA, GW, DH, DP, DM, NS, GN, Yield, RWC, Na, K, K/Na, SPAD, Tchl, and Car denote; plant height, peduncle length, flag leaf area, grain weight, number of days to heading, number of days to pollination, number of days to maturity, number of spikes per plant, number of grains per spike, grain yield per plant, relative water content, SPAD chlorophyll, total chlorophyll, and carotenoid concentration, respectively.

*, ** and ns represent significance at *p* < 0.05, *p* < 0.01, and non‐significant, respectively. The results are expressed as means ± SE.

### Gene action in the inheritance of agronomical and physiological traits

The estimates of additive, dominance, and epistatic genetic effects controlling the traits evaluated in normal and salinity-stress field conditions are given in Tables 5 and 6, respectively. In both normal and salinity stress treatments, the overall mean (m) was significant for all the traits. The six-parameter model showed that additive gene effect was the major contributing gene action in the inheritance of PH in the normal conditions. In salinity stress conditions, the additive, additive × additive, and dominance × dominance effects were the significant contributing factors in the expression of PH. In addition, there was a negative and significant dominant effect [h] contributing in PH, represents a shortening effect on the height in both conditions. Additive, dominance, and additive × additive gene effects were significant for PL in normal conditions (Table 5). In contrast, the additive and additive × additive gene interaction played a significant role in governing PL in salinity stress conditions (Table 6).

**Table 5:** Estimates of the types of gene action (gene effects) of the traits using six-parameter model under normal field conditions.

Trait	Gene effect					
	m	[d]	[h]	[i]	[j]	[l]
PH	67.71** ± 2.13	4.66* ± 0.36	-0.58* ± 0.03	4.38^ns^ ± 0.14	-2.51* ± 0.33	6.35^ns^ ± 0.16
DH	169.99** ± 3.27	-3.61* ± 0.14	13.45* ± 0.88	6.77* ± 0.07	1.55^ns^ ± 0.04	-7.31^ns^ ± 0.12
DP	174.02* ± 3.18	-6.73* ± 0.28	-4.95* ± 0.23	-7.23* ± 0.65	9.23^ns^ ± 0.48	4.14^ns^ ± 0.03
DM	213.98** ± 4.28	-3.06* ± 0.20	9.50^ns^ ± 0.68	6.61* ± 0.30	2.94* ± 0.17	-3.92^ns^ ± 0.05
PL	6.86** ± 0.48	2.05** ± 0.18	0.98* ± 0.16	1.68** ± 0.09	-0.23^ns^ ± 0.05	0.08^ns^ ± 0.01
FLA	84.72** ± 1.26	23.83* ± 0.44	47.22** ± 2.23	42.78** ± 1.08	15.18^ns^ ± 0.42	-13.65^ns^ ± 0.18
GW	3.46** ± 0.38	-0.12* ± 0.02	0.25* ± 0.02	0.24^ns^ ± 0.02	-0.13^ns^ ± 0.01	0.26*± 0.01
NS	7.48** ± 0.77	0.27* ± 0.08	0.80^ns^ ± 0.08	1.26*± 0.06	-0.30^ns^ ± 0.01	1.40* ± 0.06
GN	31.39* ± 1.17	-4.12^ns^ ± 0.48	17.74* ± 0.53	13.50^ns^ ± 0.55	3.21^ns^ ± 0.06	-4.19* ± 0.27
Yield	23.63** ± 1.28	-6.88^ns^ ± 0.17	4.17** ± 0.06	7.80^ns^ ± 0.09	4.40^ns^ ± 0.13	18.83* ± 0.36
RWC	83.45** ± 0.39	-1.53*± 0.02	0.08^ns^ ± 0.01	1.68* ± 0.11	2.55^ns^ ± 0.09	1.33^ns^ ± 0.14
SPAD	44.61** ± 1.21	-2.48* ± 0.11	12.44^ns^ ± 0.31	6.25* ± 0.03	1.22^ns^ ± 0.05	-8.02^ns^ ± 0.04
Na	0.10* ± 0.02	0.03* ± 0.01	-0.16^ns^ ± 0.01	0.01* ± 0.01	0.06^ns^ ± 0.02	0.18^ns^ ± 0.01
K	1.47** ± 0.19	0.04* ± 0.01	0.58^ns^ ± 0.01	0.33* ± 0.01	0.23^ns^ ± 0.06	-0.22^ns^ ± 0.02
K/Na	33.77** ± 1.14	11.77* ± 0.32	34.47*± 1.02	7.11* ± 0.03	-15.19^ns^ ± 0.33	-50.88* ± 1.12
Tchl	0.65** ± 0.16	-0.04* ± 0.01	0.47*± 0.04	0.14*± 0.01	-0.25* ± 0.02	-0.35^ns^ ± 0.01
Car	0.18** ± 0.02	0.02* ± 0.01	-0.08* ± 0.02	0.04* ± 0.01	-0.02* ± 0.01	0.03^ns^ ± 0.01

m, [d], [h], [i], [j], and [l] denote: mean, additive effect, dominance effect, additive × additive, additive × dominance, and dominance × dominance, respectively.

PH, PL, FLA, GW, DH, DP, DM, NS, GN, Yield, RWC, Na, K, K/Na, SPAD, Tchl, and Car represent: plant height, peduncle length, flag leaf area, grain weight, number of days to heading, number of days to pollination, number of days to maturity, number of spikes per plant, number of grains per spike, grain yield per plant, relative water content, SPAD chlorophyll, total chlorophyll, and carotenoid concentration, respectively.

*, ** and ns represent significance at *p* < 0.05, *p* < 0.01, and non‐significant, respectively. The results are expressed as means ± SE.

**Table 6:** Estimates of the types of gene action (gene effects) of the traits using six-parameter model under salinity-stress field conditions.

Trait	Gene effect					
	m	[d]	[h]	[i]	[j]	[l]
PH	64.02** ± 1.88	3.83* ± 0.14	-7.66* ± 0.37	1.48* ± 0.03	-1.59^ns^ ± 0.06	12.55* ± 0.48
DH	170.45** ± 3.01	-3.92* ± 0.09	3.23^ns^ ± 0.16	4.19* ± 0.11	-1.39^ns^ ± 0.03	0.68^ns^ ± 0.10
DP	173.01** ± 2.75	-2.84*± 0.11	6.03* ± 0.40	5.83* ± 0.32	3.68^ns^ ± 0.41	-1.07* ± 0.02
DM	208.41** ± 3.77	-2.01* ± 0.06	13.60^ns^ ± 0.19	7.12*± 0.21	-1.68^ns^ ± 0.16	-5.95^ns^ ± 0.17
PL	5.27** ± 0.34	1.77*± 0.21	2.94^ns^ ± 0.03	2.41*± 0.09	-0.32^ns^ ± 0.04	-1.09^ns^ ± 0.22
FLA	67.84* ± 0.96	11.81^ns^ ± 0.33	55.18^ns^ ± 1.17	35.96^ns^ ± 0.71	0.34^ns^ ± 0.21	-10.71^ns^ ± 0.68
GW	3.02*± 0.26	0.27^ns^ ± 0.03	0.60*± 0.02	0.34^ns^ ± 0.15	-0.32^ns^ ± 0.11	0.10^ns^ ± 0.02
NS	10.76** ± 0.30	0.76* ± 0.10	-1.74^ns^ ± 0.15	0.89* ± 0.08	-2.51^ns^ ± 0.35	3.47^ns^ ± 0.03
GN	17.39** ± 0.63	1.75^ns^ ± 0.06	34.56* ± 1.03	18.96^ns^ ± 0.31	-2.23^ns^ ± 0.24	-15.73* ± 0.53
Yield	18.63** ± 0.54	-4.09^ns^ ± 0.12	14.47** ± 0.63	6.71^ns^ ± 0.10	-2.38^ns^ ± 0.01	3.31* ± 0.27
RWC	76.28* ± 0.39	-3.41^ns^ ± 0.01	6.48^ns^ ± 0.07	0.42^ns^ ± 0.02	-1.17^ns^ ± 0.12	-1.53^ns^ ± 0.08
SPAD	48.87** ± 0.60	-3.01** ± 0.21	-5.99* ± 0.32	1.74*± 0.06	-1.63^ns^ ± 0.25	7.16*± 0.19
Na	0.12** ± 0.01	0.08* ± 0.01	-0.22^ns^ ± 0.04	0.10* ± 0.01	0.11^ns^ ± 0.001	0.25^ns^ ± 0.04
K	1.14** ± 0.15	0.04* ± 0.01	0.64^ns^ ± 0.02	0.22* ± 0.05	0.28^ns^ ± 0.01	-0.36^ns^ ± 0.08
K/Na	20.42* ± 0.61	4.33** ± 0.13	133.01** ± 2.20	32.60* ± 0.14	-44.42^ns^ ± 1.12	-102.68** ± 1.66
Tchl	0.94* ± 0.08	-0.05^ns^ ± 0.01	0.16*± 0.02	0.10^ns^ ± 0.01	-0.08* ± 0.01	-0.13^ns^ ± 0.01
Car	0.19* ± 0.02	-0.08^ns^ ± 0.01	0.13* ± 0.03	0.06^ns^ ± 0.01	-0.03*± 0.01	-0.12^ns^ ± 0.01

m, [d], [h], [i], [j], and [l] denote: mean, additive effect, dominance effect, additive×additive, additive×dominance, and dominance×dominance, respectively.

PH, PL, FLA, GW, DH, DP, DM, NS, GN, Yield, RWC, Na, K, K/Na, SPAD, Tchl, and Car denote; plant height, peduncle length, flag leaf area, grain weight, number of days to heading, number of days to pollination, number of days to maturity, number of spikes per plant, number of grains per spike, grain yield per plant, relative water content, SPAD chlorophyll, total chlorophyll, and carotenoid concentration, respectively.

*, ** and ns represent significance at *p* < 0.05, *p* < 0.01, and non‐significant, respectively. The results are expressed as means ± SE.

Additive and additive × additive were the main types of gene actions related to the inheritance of leaf K and Na concentrations in both environmental conditions. At the same time, additive, dominance, additive × additive, and dominance × dominance effects were the likely mode of gene actions in the inheritance of K/Na ratio under both conditions (Tables 5 and 6). Additive, additive × additive, and dominance × dominance effects were the major contributors to the inheritance of NS in normal conditions, while it was predominantly controlled by additive and additive × additive types of gene action in salinity treatment (Table 6). Under normal conditions, NS and GN were coordinately inherited with grain yield, while GN and yield were found to have homogeneous genetics in salinity stress conditions. The mode of inheritance of grain yield was dependent upon dominance and dominance × dominance actions of the genes in both environments.

### Heritability and components of genetic variance

The components of genetic variance (additive, dominance, and direction of dominance), average degree of dominance, broad-sense heritability (h^2^_b_), narrow-sense heritability (h^2^_n_), and for the traits in normal and salinity stress conditions are presented in Table 7 and 8, respectively. The overall comparison between additive (D) and dominance (H) variances showed that additive genetic variance has a higher contribution than dominance variance for PH, DM, PL, FLA, GW, NS, GN, RWC, SPAD, K/Na, Car traits in normal conditions. In contrast, dominance variance was larger than the additive variance for yield, DH, DP, K, Tchl. In salinity stress conditions, additive genetic variance was more significant than dominance variance for DH, DM, PL, FLA, GW, NS, K, K/Na traits. In contrast, dominance variance was more important for yield, RWC, SPAD, GN, PH, and DP. As expected, the h^2^_b_ estimates were higher than the h^2^_n_ estimates for all the traits in both environments. Moreover, both the h^2^_b_ and h^2^_n_ estimates were higher for normal (Table 7) than stress (Table 8) conditions. The h^2^_b_ ranged from 30 to 94 percent in normal and 21 to 75 percent in stress conditions. The range of h^2^_n_ was from 14 to 41 percent in normal and 1 to 38 percent in stress conditions. The h^2^_n_ estimates were higher for FLA (38%), and NS (33%) intermediate for K/Na (29%), and lower for SPAD (13%), DP (12%), PH (10%), and yield (1%) under stress conditions. The low h^2^_n_ estimates in normal conditions were 16% for K, 15% for DP, and 14% for yield. The highest h^2^_b_ values were recorded for Tchl (94%) followed by Car (83%), DP (66%), Na (60%), and K/Na (50%) in normal conditions, whereas the highest values were observed for Car (75%), Tchl (63%), Na (60%), and K/Na (48%) in saline conditions.

**Table 7:** Genetic variance components/parameters for the traits studied in normal field conditions.

				Genetic components			Heritability			
Trait	V_E_	V_P_	V_G_	D	H	F	h^2^_b_ (%)	h^2^_n_ (%)	F/√H×D	(H/D)^1/2^
PH	3.63	6.68	3.05	2.12	0.60	-0.33	46	32	-0.29	0.53
DH	2.62	6.23	3.61	1.6	1.69	-0.32	58	26	-0.19	1.03
DP	1.03	3.04	2.01	0.47	1.35	0.19	66	15	0.24	1.69
DM	3.50	5.86	2.36	1.45	0.88	-0.03	40	25	-0.03	0.78
PL	0.30	0.44	0.14	0.12	0.01	-0.01	32	27	-0.29	0.29
FLA	0.91	1.65	0.74	0.67	0.05	-0.02	45	41	-0.11	0.27
GW	0.32	0.46	0.14	0.12	0.01	-0.01	30	26	-0.29	0.29
NS	0.85	1.39	0.54	0.50	0.03	0.01	39	36	0.08	0.24
GN	4.40	7.79	3.39	2.72	0.51	0.16	44	35	0.14	0.43
Yield	3.12	4.92	1.80	0.71	1.04	0.05	37	14	0.06	1.21
RWC	3.71	7.27	3.56	1.70	1.23	0.63	49	23	0.44	0.85
SPAD	1.15	1.76	0.61	0.49	0.09	-0.03	35	28	-0.14	0.43
Na	0.02	0.05	0.03	0.01	0.01	0.01	60	20	1.00	1.00
K	0.24	0.45	0.21	0.07	0.09	-0.05	47	16	-0.63	1.13
K/Na	0.13	0.26	0.13	0.08	0.04	-0.01	50	31	-0.18	0.71
Tchl	0.01	0.17	0.16	0.05	0.09	0.02	94	29	0.30	1.34
Car	0.01	0.06	0.05	0.02	0.01	0.02	83	33	1.41	0.71

h^2^_b_, h^2^_n_, V_P_, V_G_, V_E_, D, H, F, and (H/D)^1/2^ represent broad-sense heritability, narrow-sense heritability, phenotypic variance, genotypic variance, environmental variance, additive variance, dominance variance, direction of dominance, and average degree of dominance, respectively.

PH, PL, FLA, GW, DH, DP, DM, NS, GN, Yield, RWC, Na, K, K/Na, SPAD, Tchl, and Car denote; plant height, peduncle length, flag leaf area, grain weight, number of days to heading, number of days to pollination, number of days to maturity, number of spikes per plant, number of grains per spike, grain yield per plant, relative water content, SPAD chlorophyll, total chlorophyll, and carotenoid concentration, respectively.

**Table 8:** Genetic variance components/parameters for the traits studied in field salinity stress.

				Genetic components			Heritability			
Trait	V_E_	V_P_	V_G_	D	H	F	h^2^_b_ (%)	h^2^_n_ (%)	F/√H×D	(H/D)^1/2^
PH	5.01	7.15	2.14	0.75	0.87	0.52	30	10	0.64	1.08
DH	3.79	6.48	2.69	1.62	0.89	-0.18	42	25	-0.15	0.74
DP	7.67	12.6	4.93	1.51	3.17	-0.25	39	12	-0.11	1.45
DM	4.76	6.17	1.41	1.28	0.07	-0.06	23	21	-0.20	0.23
PL	0.30	0.42	0.12	0.10	0.01	-0.01	29	24	-0.32	0.32
FLA	1.06	1.88	0.82	0.71	0.06	-0.05	44	38	-0.24	0.29
GW	0.33	0.42	0.09	0.07	0.01	-0.01	21	17	-0.38	0.38
NS	0.78	1.26	0.48	0.41	0.03	0.04	38	33	0.36	0.27
GN	4.33	6.45	2.12	0.96	1.11	-0.05	33	15	-0.05	1.08
Yield	2.83	4.23	1.40	0.04	1.28	0.08	33	1	0.35	5.66
RWC	4.06	7.62	3.56	1.29	1.71	0.56	47	17	0.38	1.15
SPAD	1.41	2.03	0.62	0.26	0.30	0.06	31	13	0.21	1.07
Na	0.02	0.05	0.03	0.01	0.01	0.01	60	20	1.00	1.00
K	0.19	0.26	0.07	0.04	0.01	-0.02	27	15	-1.00	0.50
K/Na	0.11	0.21	0.10	0.06	0.03	-0.01	48	29	-0.24	0.71
Tchl	0.03	0.08	0.05	0.02	0.02	0.01	63	25	0.50	1.00
Car	0.01	0.04	0.03	0.01	0.01	0.01	75	25	1.00	1.00

h^2^_b_, h^2^_n_, V_P_, V_G_, V_E_, D, H, F, and (H/D)^1/2^ represent broad-sense heritability, narrow-sense heritability, phenotypic variance, genotypic variance, environmental variance, additive variance, dominance variance, direction of dominance, and average degree of dominance, respectively.

PH, PL, FLA, GW, DH, DP, DM, NS, GN, Yield, RWC, Na, K, K/Na, SPAD, Tchl, and Car denote; plant height, peduncle length, flag leaf area, grain weight, number of days to heading, number of days to pollination, number of days to maturity, number of spikes per plant, number of grains per spike, grain yield per plant, relative water content, SPAD chlorophyll, total chlorophyll, and carotenoid concentration, respectively.

The significance of dominance effects relating to the additive deviations of genes is determined by the average degree of dominance [(H/D)^1/2^]. Accordingly, in this study, the ratio of (H/D)^½^ for some of the traits was lower than one, indicating the likely importance of the overdominance type of gene action in the inheritance of these traits. The direction of dominance (F) was positive for NS, RWC, Na, Tchl, Car, and yield in both environments, DP and GN in normal, and PH and SPAD in stress environment. These show the existence of more dominant alleles than recessives alleles in parents for the traits mentioned above, that showed positive F values. The negative ’F’ value of K and K/Na showed the predominance of recessive alleles governing these traits in the parents grown under saline conditions. In contrast, it was reversed in the case of Na.

## Discussion

Knowledge of genetic components of tolerance would be vital for developing salinity tolerant cultivars in wheat through its implications for the design and deployment of appropriate tools and strategies [6]. For the traits studied in the current study, (i) the assumption of significant difference (*p* < 0.01) among parental, F_1_, F_2_, BC_1_, and BC_2_ generations allows us to conduct the genetic analysis; (ii) the rejection of null-hypothesis of the additive-dominance model (three-parameter) guides us to consider an alternative model (six-parameter); and (iii) the significant mean effect “m” revealed by the six-parameter model for all the traits, supports the quantitative inheritance of the traits studied. One of the central findings from our study was that different gene actions governing the traits in the two environmental conditions (normal and salinity). Does this imply that breeding strategy for each trait should be developed per a basic gene action in the trait of interest in each of the environments? In general, the answer is “yes”, though we should expand an identical strategy to those are similar in gene action.

All the four ’A’, ’B’, ’C’, and ’D’ scales were significant for DM and yield in stress conditions. These shows that not only additive and dominance but also other types of gene action such as epistasis may likely contribute to the genetics of DM and yield in wheat when grown under saline conditions. These results are in close agreement with the recent report of Attri et al. [17] on DM and yield in three bread wheat crosses based on normal growth conditions. Yield is the most crucial agronomic trait for selecting to abiotic stress tolerance, including salinity tolerance [2]. In the current study, salinity stress caused a significant decrease in yield and significant genotypic variation for salinity tolerance, findings that are consistent with previous research [6, 14, 29].

Additive gene action was found to be one of the positive and significant genetic components in the inheritance of Na, K, and K/Na in salinity stress conditions. Therefore, recurrent selection-based breeding schemes are effective in improving salinity tolerance since we deal with a fixable component of genetic variance [26]. Similar results were found for PH, PL, and NS. These results are somehow consistent with the observations by Yao et al. [18], who showed significant additive gene effect for the PH and PL in wheat under normal environment. A significant dominance effect for yield and GN under both conditions may suggest that heterosis is an outcome of development of hybrids by crossing contrasting pure lines [5, 30]. In contrast, the negative dominance effect obtained for PH under both normal and salinity stress treatments showed that the alleles responsible for short height were dominant over the alleles representing tall plant status [31]. Indeed, the opposite signs of h and l types of gene mode actions counterbalance each other, thus resulting in decreased heterosis [17, 31]. Our data obtained from salinity-stress conditions are consistent with a recent study using a six-parameter model in wheat that reported significant duplicate epistasis for PH and yield in normal conditions [17]. Indeed, the differences in gene action types between normal and stress conditions indicate that a distinct set of genes are activated to protect the plant from stress when a plant is subjected to salinity stress [2].

Significant additive × additive effect for DP in normal conditions suggests that epistasis between alleles of different loci contributes to decreasing the DP expression [30]. The yield and GN traits were predominantly affected by dominance and epistatic gene effects. The significant effects of dominance and epistatic types of gene action in the expression of grain yield in wheat has been reported [32]. These results are also consistent with those obtained by Sharma et al. [33].

The significant effects of the additive and additive × additive that were higher than the dominance effect for NS in salinity stress conditions may reflect an appreciation of enhanced response to selection. Negative additive × additive interaction for some of the studied traits, such as DP in normal treatment, shows the potential for reducing these traits along with fixation of additive effects in the subsequent generations [34]. The slightly larger dominance genetic variance than the additive one for PH in saline conditions indicates the comparable role of both types of gene action in governing plant status.

Higher narrow-sense heritability estimates were found for K/Na ratio than either K or Na in saline conditions. In contrast, higher broad-sense heritability estimates were found for Na in both stress and normal field conditions. Together, these may imply the distinct mode of gene action in saline field environment and that K/Na is the most efficient screening criterion for salinity tolerance in wheat, as emphasized previously [12, 10]. Therefore, these findings and their twin implications strongly support that inference that selection for salinity tolerance should be considered a distinct territory for wheat breeding. One possible reason for the higher heritability of K/Na is not related to quantitative genetics per se but is rather due to the involvement of major genes. This inference has been supported in experimental research by Dashti et al. [10], and the same general point has been made from other perspectives. The new finding combines the importance of maintaining a high K^+^/Na^+^ ratio in the leaves with the established knowledge of mechanism underlying the substantial contribution of the *Kna1* locus in salinity tolerance via shoot Na^+^ exclusion in bread wheat ([2, 3, 35]). These findings are in line with the review by Benito et al. [36] who highlighted the significant of the additive effects of the genes controlling K/Na ratio and its high h^2^_n_.

## Conclusions

Over the past decades, although the knowledge of genetics and genomics has improved substantially, the additional insights would help the breeder to plan more accurate breeding tools and selection strategies. This study may provide a better understanding of the genetic architecture underlying quantitative trait variation related to salinity tolerance in wheat. Though K/Na possessed higher narrow-sense heritability than either K or Na, additive gene action is a significant contributor to the genetic components controlling Na, K, and K/Na in salinity stress conditions. The yield and GN were predominantly governed by dominance and epistatic modes of gene action. Thus, any breeding strategy for improving these traits should depend on hybrid development especially in saline environment. The findings might also have broader implications for those interested in the genetic mechanisms of salinity tolerance and in improving cultivars for tolerance to salinity in wheat.

## Supporting information

S1 Table (XLSX)

S2 Table (XLSX)

## List of abbreviations

Car: Carotenoid
FLA: Flag leaf area
DH: Number of days to heading date
DP: Number of days to pollination date
DM: Number of days to maturity date
GN: Number of grains per spike
GW: grain weight
K: Potassium
Na: Sodium
NS: Number of spikes per plant
PH: Plant height
PL: Peduncle length
RWC: Relative water content
Tchl: Total chlorophyll
Yield: Grain yield per plant
m: mean
[d]: additive effect
[h]: dominance effect
[i]: additive × additive effect
[j]: additive × dominance effect
[l]: dominance × dominance effect
